# Regulatory Framework for Supporting the Integration and Use of Biosimilars in the Private Healthcare System of the United Arab Emirates (UAE)

**DOI:** 10.7759/cureus.74581

**Published:** 2024-11-27

**Authors:** Mohamed Farghaly, Kareem A El-Fass, Nabil Amin, Shazia Qaiser, Mona Attallah, Qasim Farooq, Mohamed Badr, Sara Al Dallal, Mona Farah, Rahul Nathwani, Atheer Alansari, Ahmad Jazzar, Ashraf Reda, Martin Lee, Ahmed Abogamal, Ahmad N Fasseeh, Zoltán Kaló

**Affiliations:** 1 Health Policy, Dubai Health Care Authority, Dubai, ARE; 2 Health Economics, Syreon Middle East, Alexandria, EGY; 3 Health Policy, National General Insurance Company (PJSC), Dubai, ARE; 4 Healthcare Solutions, MedNet Global Healthcare Solutions, Dubai, ARE; 5 Health Policy, Metlife, Dubai, ARE; 6 Operations, Nextcare, Dubai, ARE; 7 Health Policy, Daman, Dubai, ARE; 8 Gastroenterology and Hepatology, Mediclinic City Hospital, Dubai, ARE; 9 Gastroenterology and Hepatology, Mohammed Bin Rashid University of Medicine and Health Sciences, Dubai, ARE; 10 Rheumatology, Mediclinic Airport Road Hospital, Dubai, ARE; 11 Gastroenterology and Hepatology, Burjeel Hospital, Abu Dhabi, ARE; 12 Dermatology, Mediclinic Welcare Hospital, Dubai, ARE; 13 Dermatology, Mohammed Bin Rashid University of Medicine and Health Sciences, Dubai, ARE; 14 Rheumatology, Mediclinic Parkview Hospital, Dubai, ARE; 15 Rheumatology, Mohammed Bin Rashid University of Medicine and Health Sciences, Dubai, ARE; 16 Rheumatology, Dr. Suliman Alhabib Hospital, Dubai, ARE; 17 Modelling, Syreon Middle East, Alexandria, EGY; 18 Health Economics, Faculty of Pharmacy, Alexandria University, Alexandria, EGY; 19 Health Economics, Semmelweis University, Center for Health Technology Assessment, Budapest, HUN; 20 Health Economics, Syreon Research Institute, Budapest, HUN

**Keywords:** biologicals, biological therapies, biosimilars, health policies, uae, united arab emirates (uae)

## Abstract

Introduction

Biologics are substantial in the treatment of different diseases; however, they can burden the healthcare systems due to their high cost. Biosimilars can help healthcare systems keep their financial sustainability and patients access to biological therapies. The research objective is to formulate a framework for integrating biosimilars in the private healthcare sector of the United Arab Emirates (UAE). This framework was based on local stakeholders’ recommendations to ensure alignment with the UAE’s healthcare market dynamics and needs.

Methods

Stakeholders from the private sector and regulators from the public sector completed a questionnaire tailored to the UAE healthcare system, based on recommendations from local stakeholders. The questionnaire encompassed five key domains: overall perceptions of biosimilars, pricing and reimbursement strategies, financing protocols, information sharing, and monitoring practices. They filled out the questionnaire during a workshop held during the 2^nd^ Conference of the Emirates Health Economics Society (EHES), conducted from 18^th^ to 20^th^ October 2022, in Jumeirah Emirates Towers, Dubai.

Results

Stakeholders showed a positive perception of biosimilars. They believed switching to biosimilars is safe, especially when it is medically supervised. Also, they advocated initiating treatment-naïve patients on the less expensive option. They also recommended that the first biosimilar should be priced at a minimum of 30% below the original product, with a preference for a discount of 50%. They also proposed that the price of the subsequent biosimilars is 80%-90% of the previous biosimilars. Health technology assessment (HTA) of biosimilars was deemed necessary by seven (58%) of the stakeholders only if the manufacturers submitted for expanding the reimbursed indication beyond the originator’s licensed indications. They recommended introducing clinical guidelines for biosimilar switching and clinical communications to show biosimilars’ effect on access to biological therapies. They advocated introducing strict financing protocols against which the prescribing patterns of the clinicians are monitored.

Conclusion

The policies proposed by the stakeholders are designed to enhance financial sustainability while optimizing spending efficiency within the Emirati healthcare system. In addition, it may enable budget reallocation to support reimbursement of high-value health technologies.

## Introduction

Biological therapies have offered therapeutic benefits to patients in different disease areas, such as cancer, multiple sclerosis, and immune-mediated inflammatory disease [1-4]. Due to their high cost, biologics pose a burden to healthcare budgets, even in high-income countries [5-7]. In the USA, biologics accounted for 37% of net spending on prescription drugs in 2017 [8], and in Europe, biologics represented 34% of medicine spending in 2021 [9].

The influx of biologics is forecast to grow. Over the period from 2022 to 2030, the biologics market worldwide is forecasted to grow annually by an average of 7.8% [10]. Thus, policymakers are required to balance maintaining access to substantial medical innovations (including biologics) and controlling their budgets [7,11-13]. This balance requires freeing up financial resources for reimbursing innovative treatments and sustaining patients' access to existing medical technologies [14].

In this context, off-patent pharmaceuticals, including biosimilars (products similar to the originator in safety and efficacy but not identical in structure [15,16]), can offer a solution to control the healthcare systems' budgets [14,17]. These cost savings can be used to finance other medical technologies. For example, a recent study showed that utilizing biosimilars led to significant savings in the national drug budget (4.6% in the Netherlands and 3.9% in Sweden) [18]. Also, in Australia, a study estimated that the use of biosimilars could have saved 24% of the medical expenditure on biologics [19]. These cost savings could be allocated to improve healthcare services for these patients or to treat more patients using the same budget [14,20]. In the United Arab Emirates (UAE), a recent study found that adopting adalimumab biosimilars can lead to savings that can reach 31.51% of the total budget allocated to autoimmune diseases [21].

The UAE is one of the countries seeking benefits from using off-patent pharmaceuticals. In 2021, the UAE gave authorization to 6,176 generics that cost approximately 60% less than their corresponding originators [22]. Abu Dhabi started to roll out a policy of dispensing generic medicines if they proved the same efficacy and safety compared to their corresponding originators [22]. Also, there are local recommendations from medical stakeholders to include biosimilars in treating some diseases, such as inflammatory bowel diseases and psoriasis [23,24].

The healthcare system in the UAE is fragmented, with Dubai and Abu Dhabi each having their own health authorities responsible for the regulatory functions [25]. In contrast, the Federal Ministry of Health oversees these functions in the remaining five emirates [25].

Private insurance is a key player in the UAE healthcare market. In Dubai, private insurance represents approximately more than 60% of the healthcare expenses in 2020 [26]. The private sector in the UAE is a key player in the healthcare market. It provides services for approximately two times the patients as the public sector [27]. Additionally, the health expenditure incurred by the private sector is expected to grow on average annually, two times more than the public sector [22]. 

Since the private sector seeks spending efficiency to cover most of the population's medical needs, biosimilars represent a major concern for the private sector and private insurance.

Although market authorization of biosimilars in the UAE is a positive move toward optimizing healthcare spending, biosimilars' policy framework is still not up to the required level for optimal utilization [28]. Policy measures are necessary for health authorities to achieve the desired benefits from the availability of biosimilars [29]. For example, a study discussing the use of infliximab biosimilars in Hungary showed that patent expiry did not manifest in increased utilization of multisource infliximab. The reason was the lack of policies regulating switching to biosimilars and promoting the use of biosimilars as first line for bionaïve patients [30].

The expanding biosimilar industry and the potential advantages of off-patent medications necessitate a policy framework for biosimilars to enable the health system to realize their benefits. Therefore, in this research, our objective is the formulation of a policy framework for biosimilars in the Emirati healthcare private sector.

This article was previously presented as an abstract at the 2023 International Society for Pharmacoeconomics and Outcomes Research (ISPOR) conference in May 9, 2023.

## Materials and methods

Overview

The study comprised two phases. First, we adapted a previously designed survey [31]. Then, we disseminated the adapted survey among stakeholders from the healthcare sector in the UAE during a workshop.

Adapting the survey

The reference survey included five domains: perception about biosimilars, policies on the pricing and reimbursements of biosimilars, financing for biosimilars, communication of information about biosimilars, and monitoring of prescribing patterns. However, the healthcare system is different in the UAE, so we adapted some of the survey questions according to the local context in the UAE, based on comments and recommendations from local stakeholders according to the needs and the context of the pharmaceutical market in the UAE. The adaptation did not cause any change in the analysis of the questionnaire; thus, the same analysis approach was adopted.

Upon discussions with local stakeholders in the UAE, some survey questions and choice options were removed as they were not relevant in the UAE setting. Local stakeholders also recommended extending the choice options for some questions to provide a broader range for responders to choose from. Finally, they recommend adding a question to assess if initiating biological treatment for all bionaïve patients should be carried out with the less costly option, and an option was added to a question about the disincentives of the more expensive medications, stating it should be positioned as a second line in the clinical practice guidelines.

Workshop

A workshop was held during the 2^nd^ Conference of the Emirates Health Economics Society (EHES), conducted from 18^th^ to 20^th^ October 2022, in Jumeirah Emirates Towers, Dubai. Stakeholders attending the workshop represented both private and public healthcare sectors. Attendees included clinical stakeholders, healthcare managers, health insurance professionals, and regulators (policymakers). During the workshop, we disseminated the adapted survey among the stakeholders.

At the beginning of the workshop, an introduction about policies on biosimilars and the structure and content of the survey were elaborated. The survey objective and domains were explained to the attendees to ensure proper comprehension. After that, the survey was administered among the stakeholders via Mentimeter® online (Mentimeter AB (publ), Stockholm, Sweden) for live, anonymous voting. The voting results were not presented until all the votes were given to avoid the influence of the previous results on those who had not voted yet. After all the stakeholders replied to the questions, the results were shown and discussed among the attendees.

## Results

Workshop results

Twelve stakeholders representing different private and public healthcare institutions in the UAE were present in the workshop. The stakeholders discussed various topics relevant to biosimilar policies. The attendees represented Dubai Health Authority (DHA) regulators, private insurance companies, and private hospitals (clinical experts). The responses of the stakeholders are shown in Table 1.

**Table 1:** Responses of the experts to the survey questions * Experts were allowed to choose multiple answers.

Domains and questions	Percentage of experts advocating (N=12)
Domain 1: The overall perception of biosimilars	
Biosimilar medicines:	
a: Do not increase patient access to biological medicines	1 (8%)
b: Have the potential to increase patient access to biological medicines	10 (84%)
c: Reduce patient access to biological medicines	1 (8%)
Biosimilar medicines:	
Are usually less effective than originators	0 (0%)
Are usually equally effective to originators	12 (100%)
Have the potential to be more effective than the originator	0 (0%)
Switching patients from biological medicines to biosimilar alternatives	
Is associated with a significantly increased risk of immunogenicity	0 (0%)
Is not associated with an increased risk of immunogenicity even if the patient is switched multiple times without medical supervision	5 (42%)
Is not associated with an increased risk of immunogenicity in the case of a single switch under medical supervision	7 (58%)
Domain 2: Pricing and reimbursement	
If there are several biological treatments with no difference in the expected therapeutic effect, the initiation of biological treatment for all naïve patients should be carried out with the less costly option	
a: Agree	12 (100%)
b: Neutral	0 (0%)
c: Disagree	0 (0%)
How much mandatory discount from the originator price would you recommend for the first biosimilar?	
No mandatory discount	0 (0%)
Mandatory 10% discount	0 (0%)
Mandatory 20% discount	0 (0%)
Mandatory 30% discount	1 (8%)
Mandatory 40% discount	2 (17%)
Mandatory 50% discount	5 (41%)
Mandatory 60% discount	2 (17%)
Mandatory 70% discount	2 (17%)
How much mandatory discount from the price of the current reference biological product would you recommend for subsequent biosimilars?	
No mandatory discount	2 (17%)
Mandatory 5% discount	0 (0%)
Mandatory 10% discount	3 (25%)
Mandatory 15% discount	4 (33%)
Mandatory 20% discount	3 (25%)
Do you suggest a revision of the biosimilar prices periodically?	
No, the price revision should only happen with the launch of a new biosimilar alternative	1 (8%)
Yes, every 6 months	0 (0%)
Yes, every 1 year	7 (58%)
Yes, every 2 years	4 (34%)
Yes, every 3 years	0 (0%)
What do you think the role of health technology assessment (HTA) would be for biosimilars?	
HTA is always required	2 (17%)
HTA is required only when the biosimilar manufacturer intends to extend the reimbursed indication (patient population) compared to the originator	7 (58%)
No HTA is required	3 (25%)
Domain 3: Financing protocols	
How the more expensive biological medicine/s should be disincentivized by healthcare payers?	
Can be prescribed only for a very limited number of patients	3 (25%)
Can be prescribed with high co-payment according to the internal price referencing	5 (41%)
Should be excluded from the formulary listings	2 (17%)
Should be moved to second-line therapy in the therapeutic guidelines	2 (17%)
Domain 4: Information sharing	
Which measures should be applied to enhance prescribers' acceptance of biosimilars? *	
Develop clinical guidelines about switching	11 (92%)
Disseminate results of cost-effectiveness studies comparing biosimilars and originators	8 (67%)
Generate real-world evidence about biosimilars	6 (50%)
Introduce financial protocol to advocate the first-line use of biosimilars for de novo patients	9 (75%)
Mandate switching patients on chronic biological treatments to cheaper biosimilar (affordable) alternatives.	6 (50%)
Share information on how biosimilar adoption decreases pharmaceutical expenditure	6 (50%)
Conduct a systematic literature review on the immunogenicity related to switching patients to biosimilar alternatives	6 (50%)
Share information on how biosimilar adoption improves patient access to biological medicines	8 (67%)
Other	1 (8%)
Which of the following aspects should be addressed with the patients to enhance their acceptance of biosimilars?*	
Better access should be explained (e.g., more treated patients, earlier initiation of biological treatment, or longer treatment duration)	7 (58%)
Explanation of equal therapeutic effect and decreased cost	7 (58%)
No need for patients' education about biosimilars	3 (25%)
Using co-payments for biological medicines with moderately higher cost	4 (33%)
Full price should be paid by patients for biological medicines with significantly higher cost	0 (0%)
Domain 5: Monitoring	
What type of monitoring is applied for prescribing patterns against the financing protocol?	
No monitoring is applied for prescribers to adhere to financing protocols.	1 (8%)
Prescribing practices are monitored against financing protocols only in case of signals of extreme prescribing practice.	1 (8%)
Prescribing practices are proactively and routinely monitored against financing protocols.	10 (84%)
In case of deviation from the financing protocol, should financial disincentives be applied to prescribers?	
Yes	7 (58%)
No	5 (42%)

Survey Domain 1: Perception About Biosimilars

Most stakeholders (10, 84%), agreed that biosimilars can increase access to biological therapies. Also, there was a consensus among the stakeholders that biosimilars are usually therapeutically equally effective to the original reference product. Some stakeholders highlighted that biosimilar regulations should ensure the same efficacy and safety between originators and biosimilars. Also, they highlighted that the originator manufacturers should not be allowed to change the manufacturing process registered during the approval process. Concerning switching from originators to biosimilars, all respondents (12, 100%) agreed there is no significant risk of immunogenicity when switching to biosimilars. Seven (58%) stakeholders considered that medically supervised single switching of biosimilars is not correlated with a higher risk of immunogenicity. In contrast, five (42%) believed multiple switches of biosimilars may not lead to an increased risk of immunogenicity. Some stakeholders underscored that the European Medicines Agency (EMA) considered there is no risk of immunogenicity even after multiple switching; however, it would be recommended to be conservative at this policy implementation phase. Also, some stakeholders highlighted that some of the physicians' concerns about switching to biosimilars may be fueled by misinformation spread by innovative companies [32].

Survey Domain 2: Pricing and Reimbursement

The survey explored three main points related to pricing: setting the price for the first biosimilar, determining prices for the subsequently introduced biosimilars, and revisiting biosimilar prices periodically. In addition, it examined two reimbursement points: initiating biosimilars in bionaïve patients and how health technology assessment (HTA) impacts policies on biosimilars.

The stakeholders recommended the price of the first biosimilar to be at least 30% less than the originator’s. However, five (41%) stakeholders advocated a 50% reduction. Moreover, 10 (83%) stakeholders recommended that the price of the subsequent biosimilars be 10%-20% less than that of the previous one. On the other hand, two (17%) stakeholders endorsed no discount to be given. The stakeholders advocating "no discount" considered the manufacturing of biosimilars more expensive than small molecules. So, high discounts might prevent the manufacturer from offering their products, which might not be profitable at these discount levels. The stakeholders supporting this opinion recommended that if discounts should be made, they should not be mandatory. On the contrary, some stakeholders advocating discounts said that if all biosimilars have the same price, this opens the door to corruption and unnecessary switching.

Regarding revising biosimilar prices, only one respondent (8%) recommended that the price should only be revised if a new biosimilar is launched. Of most stakeholders, seven (58%) suggested revising the biosimilars' prices annually, while 4 stakeholders (34%) suggested biennial revisions. During the discussion, stakeholders warned of too frequent (every six months) price revisions, which may lead to multiple unnecessary switching among the biosimilars. The Ministry of Health and Prevention (MOHP) periodically reviews all products' prices in the UAE every five years. If the price of the innovated drug is reviewed, biosimilar drugs of the re-priced innovated drug shall be reviewed, and their prices shall be accordingly reduced at the same rate [33].

Regarding the role of HTA, seven (58%) stakeholders regarded HTA as necessary only when the manufacturer requests expanding the indication (patient population) approved for reimbursement beyond the originator’s licensed indications.

There was a consensus among the stakeholders that when there are biological treatment options with no difference in the expected therapeutic effect, the initiation of biological treatment for all naïve patients should automatically be carried out with the less costly option.

Survey Domain 3: Financing Protocols

To disincentivize the use of the more expensive biologics, five (41%) stakeholders advocated high co-payment according to the internal price referencing as a policy tool, while three (25%) recommended prescribing the more expensive biologics to a very limited number of patients. Some stakeholders advocated that patients on the expensive originator biologic for a while should not be forced to switch and that this matter should be liable for a patient-physician discussion. To blend the opinions, one of the stakeholders recommended that national targets of expensive originator biologics should be determined.

Survey Domain 4: Information Sharing

Sharing information about biosimilars among the patients and prescribers was addressed since it enhances the biosimilars’ uptake into the healthcare system and the subsequent success of biosimilars' implementation.

When stakeholders were asked about strategies to improve the prescribers’ acceptance of biosimilars, they were presented with multiple choices and could select one or more. The majority (11, 92%) of votes favored the distribution of switching guidelines, nine (75%) endorsed implementing financial incentives to prescribers for using biosimilars as the first line of treatment for bionaïve patients, and eight (67%) supported sharing information highlighting the benefits of biosimilars to enhance patient access to biological therapies.

Similarly, when stakeholders were asked about strategies to improve patients’ acceptance of biosimilars, they were given multiple choices and could select one or more. Seven (58%) votes favored explaining that biosimilar use would provide more access to expensive therapies since they are less expensive and equally effective for the originator. Also, four (33%) supported applying co-payments for moderately high-cost biologics.

Survey Domain 5: Monitoring

Ten stakeholders (84%) advocated routine proactive monitoring of prescribing practice against the financing protocols. Some stakeholders advocated no routine monitoring because patients are heterogeneous, especially in the private sector, and insurance policies differ. So, they suggested monitoring can be used on aggregated data from the overall use, not individual prescribers.

In the case of deviation from financing protocols, seven (58%) stakeholders endorsed financial disincentives. Some stakeholders suggested financial incentives in case of compliance with the financing protocols.

Policy Recommendations Not Raised in the Survey

Some stakeholders suggested policy points that were not raised in the survey. There was a recommendation to limit prescribing expensive biologics only to consultants, not specialists. This recommendation was made to ensure that patients who are prescribed expensive biologics really need them. Also, clinical stakeholders were advocating switching to biosimilars by the physician but not at the pharmacy level without the consultant's approval. Finally, a unified biosimilar procurement policy was recommended to avoid multiple switching. Some hospitals procure biosimilar brands according to the rebates and discounts they get from the manufacturer; this may lead to the availability of different biosimilars at different time points, and thus, mandatory switching is made.

Policy Recommendations Summary

At the end of the workshop, the experts discussed the results and formulated policy recommendations for biosimilars in the UAE. The recommendations are illustrated in Table 2.

**Table 2:** Policy recommendations for biosimilars in the United Arab Emirates

S.No.	Pricing and reimbursement recommendations
1	Biological treatment for all naïve patients is recommended to be carried out with the less costly option.
2	It is recommended that the price of the first biosimilar is at least 30% less than the originators.
3	For subsequent biosimilars (second or later), it is recommended that the price is 10-20% less than the most recent biosimilar.
4	Prices of biosimilars are recommended to be reviewed annually or every two years.
5	Health technology assessment (HTA) is recommended to be applied to biosimilars when the manufacturer seeks to expand the reimbursed indication beyond the marketing authorization of the originator.
	Financing protocols recommendations
1	The more expensive biological medicines are recommended to be disincentivized by healthcare payers. The disincentives are recommended to include applying high co-payment according to the internal reference pricing or through prescribing to a very limited number of patients.
	Information sharing
1	Sharing information with prescribers to enhance their acceptance of biosimilars is recommended to include developing guidelines about switching between originators and biosimilars. Also, it is recommended to include how the benefits of biosimilars in enhancing patient access to biological therapies and disseminate the outcomes of cost-effectiveness studies that compare biosimilars to their originators.
2	Sharing information with patients to enhance their acceptance of biosimilars is recommended to include explaining how biosimilars can improve patients' access to biological therapy. Also, it is recommended that patients are educated about the equal therapeutic effect of biosimilars and originators and the decreased cost of biosimilars.
3	Financial protocols are recommended to be introduced to encourage using biosimilars as the first line for bio-naïve patients.
	Monitoring
1	Prescribing practices are recommended to be monitored proactively and on a routine basis against the established financing protocols.
2	If deviation from the financing protocols is found, financial disincentives are recommended to be applied for prescribers.
3	Switching of patients on originator biologics is recommended to be medically supervised.

## Discussion

The objective of healthcare policymakers is to maximize the health outcomes for their population [34] while minimizing system inefficiencies and maintaining financial sustainability. With their increasing prices, the accelerated pace of health innovation, especially in biologics, renders smooth and sustainable access to medical innovation questionable. As a result, different high-income countries-especially in Europe, have taken steps since 2006 for biosimilar adoption and formulated policy frameworks to maximize the benefit of adopting biosimilars. The healthcare stakeholders in the UAE were also interested in developing an advanced biosimilar policy framework to get headroom for innovation.

This study has reached some recommendations for the policy framework of biosimilars in the UAE through surveying stakeholders.

Generally, the perception of biosimilars is positive; stakeholders regard biosimilars as having the potential to improve access to biological therapies with comparable efficacy to the originator. This perception concords with that reported among physicians [35] and payers [36]. Such perception is substantial for adopting and using biosimilars within the health system.

The stakeholders consider that switching to biosimilars does not pose a significant risk of immunogenicity, especially when done once and medically supervised. This also concords with the evidence found in literature from controlled trials [37-40] and real-world evidence [37,39,40]. These beliefs and supporting evidence should be communicated to prescribers to alleviate their concerns regarding the safety of biosimilars, particularly in countries with a high demand for them but where their use is limited. Also, the EMA recently issued a statement that biosimilars approved in the European Union (EU) are interchangeable with their original reference product or with an equivalent biosimilar [41]. This flags the necessity for a strict and transparent approval process and quality checking of the biosimilars.

For the pricing policy of biosimilars, several stakeholders recommended that the price of the first biosimilar introduced be at least 30% less than that of the reference product. This internal reference pricing system is implemented in some countries [42,43], such as Saudi Arabia, Italy, and Switzerland [44]. In Italy, net discounts are applied for originators in the hospital sector, approaching 50% [45]. A similar survey in Egypt showed that the stakeholders recommended a 30% discount on the originator's price for the first biosimilar [31]. This discrepancy can be attributed to the higher prices of medicines in the UAE [46] compared to Egypt. Furthermore, for subsequent biosimilars, the stakeholders recommended that a 15% discount be applied compared to the immediate previous biosimilar. Such policy is implemented in some countries [47], such as Saudi Arabia [44] and Austria [43]. This policy aims to avoid unnecessary switching if all biosimilars have the same price.

The stakeholders preferred regular reviews of biosimilars' prices annually or biennially. In France, the prices of biologics and biosimilars are revisited every 18-24 months [48,49]. Currently, MOHP reviews the prices of biosimilar drugs when their innovative drugs' prices are reviewed (periodic review every five years). The unnecessarily frequent price review can be resource exhaustive and may lead to disruption in the market dynamics, which can be devastating, especially in the early phases.

The stakeholder panel regarded HTA for biosimilars as mandatory if the manufacturer seeks to expand the indication to be reimbursed beyond the reference product’s indication. There is a wide variation among countries in applying HTA to biosimilars. Some countries, such as Scotland, do not require the biosimilar to undergo the HTA process if the originator is not reimbursed [50]. In Wales, biosimilars don’t go through appraisals if the originator is already approved for the same indication and patient population, provided that the cost of the biosimilar is not more than that of the reference product [51]. Health technology assessment's role in biosimilars varies according to each country's reimbursement system and the depth of HTA implementation. In the UAE, stakeholders considered a potential role for HTA; this role may change when the HTA institutionalization process is more developed [52]. There was consensus that treatment-naïve patients should start with the less costly option. This policy tool is essential for enhancing the uptake of biosimilars and maximizing the benefits [30].

To disincentivize the use of expensive biologics, stakeholders suggested implementing high co-payments for patients who insist on receiving expensive biologics without justified need. Switzerland has adopted this strategy, where patients may face higher co-payment rates for medications when a less expensive alternative is available on the specialty list [53]. On the other hand, some countries apply a reduced co-payment on biosimilars to encourage patients to accept them [53].

Relying only on free-market forces to uptake biosimilars into the healthcare system may not be sufficient; there are increased concerns about the evidence supporting biosimilar use and their decreased efficacy. Moreover, the fear of biosimilar immunogenicity is common. Therefore, it would be necessary to share information with patients and physicians to enhance their acceptance of biosimilars.

For enhancing prescribers' acceptance of biosimilars, the strategies most advocated by stakeholders were developing switching guidelines, encouraging the use of biosimilars as first line for treatment-naïve patients through applying financing protocols, and sharing information about the benefits of biosimilars in improving patient access to biological therapies. Addressing knowledge gaps, especially for switching [35], with physicians can contribute to better uptake of biosimilars [54]. Also, different financial incentive protocols have been applied in some countries [55]. Additionally, a gain share agreement has been applied in the UK [56].

To enhance the acceptance of biosimilars among patients, stakeholders selected one or more strategies from the alternatives they were shown. The strategies most advocated by the stakeholders were explaining that biosimilars significantly enhance patients’ access to high-cost biological therapies and that although they are less expensive, they have comparable efficacy. Also, applying co-payments was recommended by some stakeholders, especially with more expensive alternatives. Explaining the benefits of biosimilars has been used in different studies with subsequently improved patient acceptance [57,58].

To ensure prescription quotas are not exceeded, the stakeholders advocated routine and proactive monitoring of the prescribing practices. Also, they recommended applying financial penalties in case a deviation is found. This aligns with policies implemented in various countries. For example, biosimilar prescriptions are monitored in Belgium and Sweden [59]. In Germany and Italy, reports indicate that prescribers face monetary penalties for not meeting prescribed quotas or targets [60,61]. Conversely, Italian physicians who achieve their targets may receive financial incentives [62].

Our study has some strong points. To our knowledge, it is the first study in the Gulf region to initiate a policy recommendations framework for biosimilars. This may open the door for more policy frameworks in the Gulf Cooperation Council (GCC) countries. Another strength is that the survey was adapted to the UAE market and ecosystem based on the recommendations of local stakeholders. This would be reflected in more practical and real-world recommendations. Our study can be limited by the low number of stakeholders answering the survey. However, the stakeholders represented key bodies involved in the Emirati private healthcare market.

## Conclusions

In conclusion, the survey revealed stakeholders' favorable views of biosimilars for improving patient access and maintaining financial sustainability in the UAE's private healthcare sector. Differential pricing was recommended to be adopted, while financing protocols should be applied to discourage the use of costly biologics and promote biosimilars for bionaïve patients.

Information sharing and education are vital for increasing biosimilar acceptance. These stakeholders’ recommendations align with policies applied in different countries. Overall, they support the cost-effective and equitable integration of biosimilars into healthcare, balancing efficacy, safety, and access.
